# The Internet of Things: Impact and Implications for Health Care Delivery

**DOI:** 10.2196/20135

**Published:** 2020-11-10

**Authors:** Jaimon T Kelly, Katrina L Campbell, Enying Gong, Paul Scuffham

**Affiliations:** 1 Menzies Health Institute Queensland Griffith University Gold Coast Australia; 2 Centre of Applied Health Economics Griffith University Brisbane Australia; 3 Metro North Hospital and Health Service Brisbane Australia; 4 School of Population and Global Health The University of Melbourne Melbourne Australia

**Keywords:** Internet of Things, digital health, smartphone, delivery of health care, mobile phone

## Abstract

The Internet of Things (IoT) is a system of wireless, interrelated, and connected digital devices that can collect, send, and store data over a network without requiring human-to-human or human-to-computer interaction. The IoT promises many benefits to streamlining and enhancing health care delivery to proactively predict health issues and diagnose, treat, and monitor patients both in and out of the hospital. Worldwide, government leaders and decision makers are implementing policies to deliver health care services using technology and more so in response to the novel COVID-19 pandemic. It is now becoming increasingly important to understand how established and emerging IoT technologies can support health systems to deliver safe and effective care. The aim of this viewpoint paper is to provide an overview of the current IoT technology in health care, outline how IoT devices are improving health service delivery, and outline how IoT technology can affect and disrupt global health care in the next decade. The potential of IoT-based health care is expanded upon to theorize how IoT can improve the accessibility of preventative public health services and transition our current secondary and tertiary health care to be a more proactive, continuous, and coordinated system. Finally, this paper will deal with the potential issues that IoT-based health care generates, barriers to market adoption from health care professionals and patients alike, confidence and acceptability, privacy and security, interoperability, standardization and remuneration, data storage, and control and ownership. Corresponding enablers of IoT in current health care will rely on policy support, cybersecurity-focused guidelines, careful strategic planning, and transparent policies within health care organizations. IoT-based health care has great potential to improve the efficiency of the health system and improve population health.

## Introduction

The challenges presented by an aging population with multiple chronic conditions are ubiquitous worldwide [1]. The medical, lifestyle, and personal health needs across aging populations will continue to place a burden on health care resources. Meeting these challenges requires a focus on empowering populations to self-manage their health through health innovation to improve well-being and attenuate health resource burden [2].

## Background of Digital Devices and the Internet of Things

Entering the 2020 decade, more devices are connected to the internet than ever before, and this will continue to grow at a rapid trajectory. Worldwide, more than 21 billion devices have been estimated to be connected to the internet in 2020, which is 5 times the number of devices 4 years prior [3]. The Internet of Things (IoT) can be defined in its simplest scenario as a network that connects uniquely identifiable devices (or *things*) to the internet, enabling them to collect, send, store, and receive data [4]. From a health care perspective, IoT can be considered as any device that can collect health-related data from individuals, including computing devices, mobile phones, smart bands and wearables, digital medications, implantable surgical devices, or other portable devices, which can measure health data and connect to the internet [5].

The growth of IoT technology has driven interest in a wide range of health practices to improve population health more specifically [6]. Recent reviews have overviewed the various services and applications of IoT in health care (eg, eHealth, mobile health [mHealth], ambient assisted living, semantic devices, wearable devices and smartphones, and community-based health care) [5,7]. These services have been detailed extensively and can have many applications across single condition and cluster condition management, including, for example, the ability to track and monitor health progress remotely by health care professionals, improve self-management of chronic conditions, assist in the early detection of abnormalities, fast-track symptom identification and clinical diagnoses, deliver early intervention, and improve adherence to prescriptions [8]. These applications can make better use of health care resources and provide quality and low-cost medical care.

## Health Systems Are Changing

With the 2020 public health response to the novel COVID-19 pandemic to effectively shut down traditional modes of health service delivery worldwide, efforts to reduce implementation barriers to technology-supported health delivery highlight the potential to reframe traditional models of care into virtual and distance modalities [9]. In response, many countries have successfully implemented technology-supported services to maintain health care practices and social distancing [10]. As global leaders consider policies that potentially provide more access to technology-supported health services in response to (and considerations post) the current COVID-19 crisis, it is becoming increasingly important to understand how established and emerging IoT technologies can support health systems to deliver safe and effective care in either a complementary or an alternative way during times of crisis or health epidemics [11].

This viewpoint paper will overview current technologies in health care, outline how IoT devices are improving health service delivery, and outline how IoT technologies can affect global health care in the next decade. This viewpoint paper also overviews how the disruption in health care from IoT can lead to improved access and equitable primary, secondary, and tertiary smart health care, which is more proactive, continuous, and coordinated.

## IoT-Based Health Care Architecture

The architecture of IoT in health care delivery essentially consists of 3 basic layers [12]: (1) the perception layer, (2) the network layer, and (3) the application layer. It is not our intention to extensively detail these layers; however, a summary and the related health implications are provided in the following sections.

### Perception Layer: Sensing Systems That Collect Data

Perception and identification technologies are the foundation of IoT. Sensors are devices that can perceive changes in an environment and can include, for example, radio frequency identification (RFID), infrared sensors, cameras, GPS, medical sensors, and smart device sensors. These sensors allow for comprehensive perception through object recognition, location recognition, and geographic recognition and can convert this information to digital signals, which is more convenient for network transmission [12,13]. Sensor technologies allow for treatments to be monitored in real time and facilitate the acquisition of a multitude of physiological parameters about a patient so that diagnoses and high-quality treatment can be fast-tracked. There are many examples of potentially lifesaving IoT sensor devices; however, not all devices are clinically tested or have been proved to be safe or effective. A summary of IoT devices that may support and improve health service delivery is provided in Multimedia Appendix 1 [14-47].

### Network Layer: Data Communication and Storage

The network level of IoT technologies includes wired and wireless networks, which communicate and store processed (layer 1) information either locally or at a centralized location. Communication between things can occur over low, medium, and high frequencies, the latter being the predominant focus of IoT. These include short-range communication technologies, such as RFID, wireless sensor networks, Bluetooth, Zigbee, low-power Wi-Fi, and global system for mobile communications [12]. High-frequency fourth-generation (4G) cellular networks have seen even more communication potential, and evolving 5G networks are becoming more readily available and are expected to be a major driver of the growth of IoT applications for health care, with the potential to provide reliable connection up to thousands of devices at the same time [48].

Communicated data are stored locally (often decentralized) or sent to a centralized cloud server. Cloud-based computing to support the delivery of health services has many benefits, as it is ubiquitous, flexible, and scalable in terms of data acquisition, storage, and transmission between devices connected to the cloud [49]. The use of the cloud can be foreseen to support data-intensive electronic medical records (EMRs), patient portals, medical IoT devices (which can include smartphone apps), and the big data analytics driving decision support systems and therapeutic strategies [5]. However, with more cloud apps entering the health market, it is just as important that an evidence base supports its effectiveness and safety and can deal with the security of health data and the reliability and transparency of that data by third parties. Furthermore, it has been suggested that centralized cloud storage will present issues in the future to users, such as excessive data accumulation and latency because of the distance between IoT devices and data centers.

Decentralized data processing and networking approaches may improve the scalability of IoT in health care. Edge cloud is a newer cloud computing concept that allows IoT sensors and network gateways to process and analyze data themselves (ie, at the *edge*) in a decentralized fashion, reducing the amount of data required to be communicated and managed at a centralized location [12,50]. Similarly, blockchain storage uses a decentralized approach to data storage, creating independent blocks containing individual sets of information, which forms a dependent link in a collective block, which in turn creates a network regulated by patients rather than a third party [51]. There are examples of platforms engineering blockchain for medical practice already [51,52]; however, research on edge cloud and blockchains in health care is still limited and is an important area for future research.

### Application Layer

The application layer interprets and applies data and is responsible for delivering application-specific services to the user [12]. Some of the most promising medical applications that IoT provides are through artificial intelligence (AI). The scientific applications of AI have proliferated, including image analysis, text recognition with natural language processing, drug activity design, and prediction of gene mutation expression [53]. AI has the capability to read available EMR data, including medical history, physical, laboratory, imaging, and medications, and contextualize these data to generate treatment and/or diagnosis decisions and/or possibilities. For example, IBM Watson uses AI to read both structured and unstructured text in the EMR, read images to highlight primary and incidental findings, and compile relevant medical literature in response to clinical queries [54].

IoT-based health care and use of deep machine learning can assist health professionals in seeing the unseeable and providing new and enhanced diagnostic capability. Although diagnostic confidence may never reach 100%, combining machines and clinician expertise reliably enhances system performance. For example, compared with the diagnostic evaluation by 54 ophthalmologists and senior residents, applying AI to retinal images improved the detection and grading of diabetic retinopathy and macular edema, achieving high specificities (98%) and sensitivities (90%) [55]. AI and deep learning can also optimize disease management, can provide big data and analysis generated from mHealth apps and IoT devices, and are starting to see adoption in health care [56]. Some examples of this include predicting risk, future medical outcomes, and care decisions in diabetes and mental health [57] and predicting the progression of congestive heart failure [58,59], bone disease [60], Alzheimer disease [61], benign and malignant tumor classification [62,63], and cardiac arrhythmias [64].

### Expanding the Functions and Scope of IoT to Provide Smart Health Care

IoT is an infrastructure that enables smart health services to operate. When health data are collected by IoT sensors, communicated, and stored, this enables data analytics and smart health care, which can improve risk factor identification, disease diagnoses, treatment, and remote monitoring and empower people to self-manage.

Smart health care services make use of advancements in information technologies, such as IoT, big data analytics, cloud computing, AI, and deep machine learning, to transform traditional health care delivery to be a more efficient, convenient, and a more personalized system [65]. Current developments in information computer technologies have allowed the development of health care solutions with more intelligent prediction capabilities both in and out of the hospital. We are seeing the use of virtual models to transfer care provided in hospitals to the home through the use of sensors and devices that allow remote review and monitoring of patients in their homes or treated in hospitals and creates a continuum among these through cloud access [7]. More recently, the 2020 public health efforts around the world to mitigate the spread of COVID-19 have (at least temporarily) led governments and policy makers to remove implementation and remuneration barriers to enable health care professionals to use virtual models of care for people who need it [9]. IoT also provides the opportunity to improve the quality and efficiency of the entire ecosystem of service delivery, including hospital management, medical asset management, monitoring of the workflow of staff, and optimization of medical resources based on patient flow [66,67].

## How IoT Can Improve Health Service Delivery

### Primary Health Care Becoming More Accessible

A focus on disease prevention must become a priority this decade, as the burden of disease attributable to modifiable risk factors is greater than ever before [1,68]. IoT in health care has the potential to improve population health and transition our health care model to a true hybrid model of primary, secondary, and tertiary care, where the health system can use its existing workforce in new and more efficient ways. Transforming health delivery in this way is crucial to improving self-management for people with chronic conditions, as even among high health care users, more than 90% of lifestyle self-management is done by patients themselves, outside of hospitals, and in clinical settings [69,70].

There is a clear public demand for easy-to-access health information. For example, in a 2015 US survey, 58% (931/1604) of smartphone users downloaded a health-related app for their lifestyle self-management [71]. AI has also driven the availability of point-of-care health information, such as chatbots (or AI doctors), which can deliver lifestyle and medical advice. Examples of these established AI *bots* are Woebot, Your.Md, Babylon, and HealthTap, where a patient can input their symptoms and advice is generated instantly [72]. However, more than half of the most highly rated apps make medical claims that are not approved [73], with no formal process of approving apps or informing consumer choice [74], and much remains to be done to understand the potential of chatbots to improve health. Therefore, a reliable digital health evidence base is essential [75]. If health professionals have evidence-based digital resources, devices, and mobile apps readily at their disposal, digital prescriptions could become an enabler of wider adoption of IoT in health care and facilitate a wider population focus on disease prevention.

At the individual level, IoT offers the opportunity to link and potentially learn from nonhealth IoT technologies to monitor daily activities, provide support with information, and promote behavior changes (Multimedia Appendix 2). In addition, IoT and data linkage create great potential of transparent, evidence-based decision making, which may be able to drive the shift of disease patterns and increase the well-being of citizens at scale. The integration of urban infrastructures, IoT technologies, and cloud computing allows the collection and analysis of a vast quantity of different human and non–human-related data. These data could provide valuable information about population-level surveillance in diseases and accidents, risk factors, and environmental conditions [76], which is difficult to collect through the traditional human-reported disease surveillance system and can be of particular benefit in pandemic responses [77]. For example, in Taiwan, big data analytics applied to electronic data (GPS, closed-circuit television surveillance, and credit card payments) in the community and personal mobile data have been effectively used to contact trace, communicate, and isolate potential contacts during the global COVID-19 pandemic [78]. Through IoT and data linkage, decision makers are likely to be able to make evidence-based decisions in promoting healthy social and built environments, safe transportation systems, high-quality public services, and smart health care and emergency response systems [76,79,80].

### Secondary and Tertiary Health Care That Is Proactive, Continuous, and Coordinated

An IoT-based health care system enables the overall health care systems to move past a traditional model of service delivery, which is often reactive, intermittent, and uncoordinated, to a more proactive, continuous, and coordinated approach [81]. Such an approach is favorable because it offers the opportunity to provide high-quality care that is less invasive and appealing to patients and health care professionals. This change in the health care system landscape is also highly appealing for policy makers because it can greatly enhance the efficiency (and subsequently reduce resource use) of the health system [82] and also provide the health system flexibility to shift its models of care and delivery of services as required on an individual or population-wide basis. Multimedia Appendix 3 summarizes 7 examples of how IoT can improve the coordination of health services and likely improve our health system efficiency.

## Enablers and Barriers to Address for IoT-Based Health Care

### Enablers

#### Policy Support

Policy support is one of the most important environmental enablers of IoT. Many countries already have policies in place for eHealth (eg, web-based and software programs to deliver health services) [83,84] and either have or are in the process of developing policies for IoT infrastructure, investment, and/or implementation in health care. For example, China, India, Indonesia, Japan, Malaysia, the Philippines, Singapore, Thailand, the European Union, the United States, and Vietnam currently have relevant policies in place for IoT [85]. Australia is also in the process of establishing a policy for IoT development and investment [86].

#### Technology That Is Accessible and Easy to Use

The ubiquitous nature of technology means that consumers and health care professionals have greater access to digital resources than ever before [87]. However, it is also important for health systems to be aware of the inequities that may eventuate from the widespread implementation of IoT for health care, including individuals who may not be able to afford or access technology hardware or reliable internet services because of geographic location or financial disadvantage. Similarly, if individuals do not perceive the technology as *user friendly*, experience poor connections, or do not feel the initiative has been designed in consultation with them (both patients and health professionals), then this often results in frustration and reluctance to use such services [88,89].

#### Cybersecurity-Focused Guidelines for Robust and Resilient Market Adoption

Cyber risk is a major obstacle to the broad adoption of IoT [90]. The privacy of patients must be ensured to prevent unauthorized identification and tracking. From this perspective, the higher the level of autonomy and intelligence of the things, the more the challenges for the protection of identities and privacy.

### Barriers

#### Confidence and Acceptability

There is a gap in public awareness and understanding of data safety in cloud-stored health information. This is of concern, as it is the single biggest threat to the adoption of IoT from a societal perspective. The premise of IoT is clear to society; however, what is not clear to people is the actual value that IoT delivers to them personally from a health care perspective [91,92]. The potential threat of breached confidentiality may never go away; however, the perceived value to consumers needs to outweigh these concerns to confidently engage with IoT-supported health infrastructure [90]. The confidence and acceptability of IoT by health care professionals are similarly important. There is a diverse range of factors that affect clinicians’ acceptability of technology-supported programs, including the characteristics of the technology (eg, accuracy, compatibility with usual systems, and ease of use), individual’s attitudes and knowledge (eg, familiarity and impact on professional security), external factors (eg, patient and health professional interaction), and organization readiness (eg, training and reimbursement) [93].

#### Privacy and Security

IoT might allow opportunities for cyberattacks and for personal data to be collected inappropriately. IoT-based applications are vulnerable to cyberattacks for 2 basic reasons: (1) most of the communications are wireless, which makes eavesdropping very easy; and (2) most of the IoT components are characterized by low energy, and therefore, they can hardly implement complex schemes on their own to ensure security. The National Institute of Standards and Technology has recently released a draft security guide and recommendations for IoT devices, which will see an emphasis on data security in IoT devices [94]; however, whether such a guideline can or will be enforced across IoT health devices is unclear. 

#### Data Storage, Control, and Ownership

To move forward in IoT-based health care, transparency and enforced codes of practice regarding where centralized cloud data are stored and who owns the data, needs to be considered For example, does the data host have viewing rights to someone’s data and are these data completely controlled by individuals or are they never deleted from the cloud, despite a user’s request? Another important consideration is the sharing of data across states or territories and internationally. Privacy, security, and confidentiality of data control and storage should be federally enforced, but international hosts and suppliers may not be required to follow any such code. Therefore, the use of these platforms requires strategic planning and transparent guidelines to develop and implement robust IoT-based health care policies and models of care.

#### Interoperability and Standardization Protocols

Issues around the interoperability and standardization of IoT and health care systems are a big threat to the wider adoption of IoT for health care systems. Lack of standardization threatens the development of IoT in the health setting context, as the industry and manufacturers are yet to reach a consensus regarding wireless communication protocols and standards for machine-to-machine communication. Without a unified, standardized, and interoperable system, the adoption of IoT into health care will be greatly hindered and is unlikely to have international reach [95]. Semantic interoperability in IoT is a necessary condition for big data techniques to support decision-making processes [96]. It is increasingly common for each new technology startup, device, or system manufacturer to define their own specific architecture, protocols, and data formats, which are unable to communicate with the health care environment unless they are appreciably redeveloped or adapted to interoperate with hospital IoT platforms [96]. This creates *Vertical Silos* [97], which demands the development of new features for granting interoperability between different systems. The future and full potential of IoT-enabled health care relies on addressing interoperability, of which some frameworks do exist [98]. Achieving interoperability across IoT platforms can provide a safer, more accessible, productive, and satisfying experience for clinicians and patients alike.

#### Remuneration

Finally, remuneration for technology-assisted health care has historically been challenging [99] and differs appreciably across different countries. This is likely to be even more complex for IoT-delivered health care, where reimbursement considerations have not been established (and this is unlikely until the abovementioned points are addressed). As international health systems establish robust policies and guidelines on cybersecurity and address the issues surrounding interoperability and standardization protocols, reimbursement and regulatory considerations across single-payer and multipayer systems should become a key priority to ensuring successful, effective, and cost-effective IoT health care models can be implemented in practice.

## Conclusions

From this viewpoint, the potential of IoT is summarized as a growing area of research in health care. These developments provide a great opportunity for health care systems to proactively predict health issues and diagnose, treat, and monitor patients both in and out of the hospital. As the adoption of technology-supported health services increases to enable health systems to deliver flexible models of care, an increasing number of traditional health service delivery practices will be complemented or replaced through IoT. However, the implementation of IoT in health care will rely on a clear and robust code of practice for the management of data, privacy, confidentiality, and cybersecurity concerning the supply and use of IoT devices in health care. There are still important gaps for future research to address, which relate to the IoT technology itself, the health system, and the users of IoT technology. Specific future research on IoT technology needs to address how IoT devices can be designed with standardized protocols and interoperability with international and cross-state health systems. More research is also needed on the efficiency of blockchain storage compared with centralized cloud-based storage solutions in the context of IoT-supported health care delivery. From a health system perspective, there is a need for clinical guidelines on digital health prescriptions and robust policy regarding remuneration for primary and secondary care services provided through IoT. Finally, more research is needed to determine the acceptability and digital literacy of consumers and clinicians in the context of using IoT to improve the delivery and overall experience of health care. Although this viewpoint is a summary of selected literature only and not based on an exhaustive systematic review of the literature, we believe that addressing these areas for future research will go a long way to enable a wider uptake of IoT, which can ultimately save health care dollars and improve patient-centered care.

## Appendix

Multimedia Appendix 1 Examples of Internet of Things devices that can support health service delivery.

Multimedia Appendix 2 Examples of how smart homes can improve health care delivery.

Multimedia Appendix 3 Scenarios where Internet of Things can be used to improve health system efficiency.

## Abbreviations

AI: artificial intelligence
EMR: electronic medical record
IoT: Internet of Things
mHealth: mobile health
RFID: radio frequency identification
