# Supplementary material for: The Internet of Things: Impact and Implications for Health Care Delivery
Source: J Med Internet Res. 2020 Nov 10;22(11):e20135. doi: 10.2196/20135 (PMC7685921; doi:10.2196/20135)
Supplement: Multimedia Appendix 1 [file jmir_v22i11e20135_app1.docx]

**Multimedia appendix**

This is a Multimedia Appendix to a full manuscript published in the J Med Internet Res.

**Multimedia Table 1:** Examples of Internet of Things devices that can support health service delivery

| IoT^a^ device | Brief description | Use and comments |
| --- | --- | --- |
| Bluetooth-enabled inhalers | Use a Bluetooth sensor, mobile app, predictive analytics, and feedback | - Used to help patients with asthma and their health care team better understand and control respiratory conditions. Research has shown these IoT devices to improve health care utilization and reduction in inhaler use through better self-management and symptom identification [14,15]. |
| Vital sign patches | Designed primarily to wirelessly track and monitor heart rate, respiration rate, temperature, steps counts, sleep cycle, stress levels, and falls or incapacitation | - One pilot trial in patients following discharge from emergency (with infection or exacerbation of heart failure, chronic obstructive pulmonary disease, or asthma) demonstrated fewer adverse events in the intervention and two-thirds lower health care costs through using the device compared with usual care [16]. - Vital sign patches are still being tested for their effectiveness and have further applications, for example, to monitor and detect changes in hypertension, type 2 diabetes mellitus, sleep apnea, asthma, and exacerbation of chronic obstructive pulmonary disease or heart failure. |
| Digital (smart) medications | An ingestible sensor (microfabricated sensor made from copper, magnesium, and silicon, in minute quantities), which communicates with an external body sensor such as a wearable sensor patch | - The smart medication interacts with a wearable patch, and wireless communicates this with a mobile app or web portal. Information is stored on the cloud and is used to measure medication adherence, absorption, activity, and heart rate. The mobile app can also be used to prompt the user to take their prescribed medication as scheduled and share information with family members or carers [17]. - Examples in pilot studies include assisting appropriate treatment for uncontrolled hypertension and diagnosing asymptomatic diseases [18]. |
| Therapeutic extended reality | Augmented reality, mixed reality, and virtual reality can visualize data collected from sensors that are part of the IoT. These create a sense of being transported into life-like, three-dimensional worlds and can be applied as an innovative treatment modality to manage a broad range of health conditions. | - Virtual reality has become increasingly portable, immersive, and vivid, which has enabled the technology to be used in a broad range of inpatient and outpatient applications [19,20]. - Augmented reality (superimposed computer images to manipulate the users’ view of the real world) and mixed reality (combined real and virtual environments) applications have been suggested to outperform traditional service methods in many clinical areas of health care, including acquisition of anatomy knowledge, anesthesia, and central vein catheterization [21]. Other virtual reality applications have been applied in mental health and anxiety disorders, stroke, and pain management [22-26] and have even been suggested to assist in obesity management and prevention [27]. |
| Wearables | Technological infrastructure worn by the user that interconnects wearable technology with wearable sensors through wireless connections [42] | - Wearable devices are diverse and evolving in a demanding market. The prudent question is, “Will wearable devices just be a peripheral for a smartphone, or is there a more important role for them as part of IoT-based healthcare?” - Examples of wearables currently used include continuous glucose monitors and smart insulin pens (which track dose, time, and recommend the correct type of insulin to use) [28]; loneliness detectors [29]; sleep trackers; smartwatches and Fitbits (to track activity, heart rate, and sleep patterns); fall detectors, iFall (a wearable accelerometer that communications with a smartphone and the cloud) to detect and respond to patient falls [30]; wireless electrocardiogram monitors [31]; and wearable blood pressure monitors [32]. |
| Smart voice assistants (conversation agents) | Installed in the home setting to provide support to users through conversations (eg, Amazon, Alexa, and Google Home) | - The hand-free feature of smart voice assistants creates unique benefits to certain population groups, for example, the older, disabled, or people with poor technology literacy, by removing barriers in typing on a smartphone or keyboard [33]. Conversation agents also play the central role in the smart home ecosystem that integrates various IoT devices. - The interactive conversations with the smart voice assistants enable a patient-centered and engaged approach, which add extra values to patients with empowerment and more control of their health [34]. The types of conversation agents used in the health setting have included apps, chatbots, SMS, web browser, Windows computer app, and telephone [34]. - Conversation agents can provide answers to a specific set of health-related questions without human contact, guide self-management activities via coaching lifestyle changes, and can collect data for screening and remote patient monitoring [35]. Some examples where conversation agents have been used to manage health conditions include depression and anxiety; autism; sexual, substance, and physical harassment; language impaired; obstructive sleep apnea; hypertension; breast cancer; type 2 diabetes; and pain management [34]. - Several industry-initiated projects have applied AI^b^ algorithms and decision trees to enable conversation agents to fulfill more complex health requests and integrated into health care delivery. For example, CardioCube supports individuals with cardiovascular diseases to complete follow-up visits, medication refills, and service referrals [36]. |
| Social robots | AI system designed to interact with humans through social rules applied to its role [37] | - In the hospital setting, robots are used to provide information to help people navigate within hospitals, detect abnormal actions, and collect patient data [38,39]. - In the home, health robots can be applied to support the detection of unhealthy behaviors, manage medication use, and assist in rehabilitation therapies [40-43]. - A systematic review found 6 of 7 studies to improve psychological well-being, finding significant improvements in depressive symptoms in 3 studies and trends toward improvements in 3 studies [37]. |
| Continuous glucose monitors and smart insulin pens | Track dose and time and recommend the correct type of insulin dosage | - These IoT compatible devices can help both patients and clinicians improve insulin administration adherence and medication errors to ultimately improve blood glucose control and management [28]. |
| Smart cameras | Smartphone cameras that can capture changes in the environment | - Smart cameras are usually associated with a smartphone and download data to an app or web portal. Examples of smart cameras to assist health care delivery include wound analysis in patients with diabetes [44], to monitor dermatitis and skin conditions [45,46] and heart rates, and to monitor tear film buildup in dry eye disease [47]. |

^a^IoT: internet of things.

^b^AI: artificial intelligence.
