# Supplementary material for: The Internet of Things: Impact and Implications for Health Care Delivery
Source: J Med Internet Res. 2020 Nov 10;22(11):e20135. doi: 10.2196/20135 (PMC7685921; doi:10.2196/20135)
Supplement: Multimedia Appendix 2 [file jmir_v22i11e20135_app2.docx]

**Multimedia appendix**

This is a Multimedia Appendix to a full manuscript published in the J Med Internet Res.

**Multimedia Table 2:** Examples of how smart homes can improve healthcare delivery

| **Short case 1** | Smart homes offer the user to control anything that can communicate through a wireless internet network through their smart device, including lighting, power, appliances, security, entertainment and security. A person wearing a smartwatch can wake up in the morning, their watch triggers the kettle to boil and the lights to turn on – the person takes milk out of the fridge and uses the remainder. The fridge notices the milk is not placed back in the fridge within 3 minutes which triggers the user’s smartphone to inform it whether they are out of milk; alternatively, when the milk is placed back in the fridge a sensor detects the remainder and use rate. The user has a conversation agent which confirms this and then milk is added to an online shopping list, purchased online and delivered to the door the next morning. |
| --- | --- |
| **Short case 2** | In an aged care setting, a patient with frailty has been discharged from hospital back into their independent living unit. The patient was discharged with a digital prescription and wears a wearable smart device to detect their heart rate and respiratory rate. This device detects when the patient moves between rooms and communicates with the smart home to automatically turn on the lights to avoid any risk of falling – this detail is captured and stored to assist in home assessments of activity of daily living, if required. If the patient has a fall, the sensor detects no movement which triggers the App to send an SOS call to emergency and an ambulance is on the scene within 20 minutes. The data stored on the cloud relating to the patient’s movements and room use throughout the day is used to inform a home assessment and digital prescription to reduce the likelihood of a subsequent fall. |
