# Supplementary material for: The Internet of Things: Impact and Implications for Health Care Delivery
Source: J Med Internet Res. 2020 Nov 10;22(11):e20135. doi: 10.2196/20135 (PMC7685921; doi:10.2196/20135)
Supplement: Multimedia Appendix 3 [file jmir_v22i11e20135_app3.docx]

**Multimedia appendix**

This is a Multimedia Appendix to a full manuscript published in the J Med Internet Res.

**Multimedia Table 3:** Scenarios where IoT can be used to improve health system efficiency

| **Scenario 1** | IoT connects the patient and their care provider, outside of the hospital and in the patient’s home to ‘treat’ / ‘monitor’ – this can save health expenditure and improve the delivery of patient-centered care |
| --- | --- |
| **Scenario 2** | IoT encourages individual self-monitoring and data-driven health decisions. For example, self-monitored data is uploaded to the cloud, and continuously fed-back to the health service. No action is needed unless a ‘trigger’ alert (generated through data analysis) which can be used to prioritize review appointments in clinic |
| **Scenario 3** | IoT encourages people to seek health support when they need it through continuous alerts and feedback provided at their fingertips – all enabled through IoT. A fully connected IoT-healthcare system could provide the patient with information on who, when and where health professional help is available |
| **Scenario 4** | Utilizing sensor-based screening and assessment devices through IoT in the community can reduce resource pressure on the hospitals environment and turn this information into an electronic flow of information |
| **Scenario 5** | IoT can improve the management of clinical workloads and resource allocations which can allow healthcare systems to effectively prioritize those patients who have the highest need for medical services |
| **Scenario 6** | Using IoT to increase point-of-care testing may reduce the time of diagnosis and reduce the need for sending samples away to be tested – which usually take many days |
| **Scenario 7** | Helping health practitioners understand the status of their patients quickly, easily and effectively |
